# Acceptability of mindfulness from the perspective of stroke survivors and caregivers: a qualitative study

**DOI:** 10.1186/s40814-018-0244-1

**Published:** 2018-02-26

**Authors:** Bhautesh Dinesh Jani, Robert Simpson, Maggie Lawrence, Sharon Simpson, Stewart W. Mercer

**Affiliations:** 1 General Practice and Primary Care, Institute of Health and Wellbeing, University of Glasgow, 1 Horselethill Road, Glasgow, Scotland G12 9LX UK; 2 School of Health and Life Sciences, Glasgow Caledonian University, Glasgow, G4 0BA UK

**Keywords:** Mindfulness, Stroke survivors, MBSR, Post-stroke depression

## Abstract

**Background:**

Depression is very common among stroke survivors with estimated prevalence rates of approximately 33% among stroke survivors, but treatment options are limited. Mindfulness-Based Stress Reduction (MBSR) is an effective treatment for depression generally, but benefits in stroke patients are unclear. The aim of this study was to determine the feasibility of delivering MBSR to stroke survivors and their caregivers in the community. We conducted a study to gain views of MBSR as a potential treatment option among stroke survivors and their caregivers in the community.

**Methods:**

Participants were recruited from an urban community in Scotland (UK) using newspaper adverts, social media and support groups run by health charities. A 2-h MBSR taster session was delivered by two experienced mindfulness instructors, followed by focus group sessions with all participants on their user experience and suggestions for MBSR modifications for stroke survivors. The focus group sessions were audio recorded and transcribed verbatim. Transcript data were analysed thematically using the framework approach.

**Results:**

The study sample consisted of 28 participants (16 females); there were 21 stroke survivors (11 females) and 7 caregivers (5 females). The median age for participants was 60 years.

Most participants described the MBSR taster session as a positive experience. The main challenge reported was trying to maintain focus and concentration throughout the MBSR session. Some participants expressed reservations about the duration of standard mindfulness course sessions, suggesting a preference for shorter sessions. The potential for achieving better control over negative thoughts and emotions was viewed as a potential facilitator for future MBSR participation. Participants suggested having an orientation session prior to starting an 8-week course as a means of developing familiarity with the MBSR instructor and other participants.

**Conclusion:**

It was feasible to recruit 21 stroke survivors and 7 caregivers for MBSR taster sessions in the community. A shorter MBSR session and an orientation session prior to the full course are suggestions for potential MBSR modifications for stroke survivors, which needs further research and evaluation.

**Electronic supplementary material:**

The online version of this article (10.1186/s40814-018-0244-1) contains supplementary material, which is available to authorized users.

## Background

Stroke is a major cause of death and disability worldwide [1–5]. The stroke mortality rate varies among different countries, with relatively low mortality reported in middle- and high-income countries [1]. An improvement in stroke-related survival rate has been reported in the UK and USA, attributed to improved primary care management of stroke-related risk factors [2, 3]. A global estimate suggests that the number of stroke survivors is likely to rise to 77 million people by 2030 [6]. The prevalence of depression is reported to be very high among stroke survivors and has been associated with adverse clinical outcomes.

A meta-analysis published in 2005 reported a pooled estimate of 33% for the prevalence of depression in stroke survivors [7]. A review published in 2010 reported an overall prevalence rate of 21.7% (range 6 to 40%) for post-stroke major depressive disorder (MDD) and 19.5% (range 8 to 44%) for post-stroke minor depression—DSM-IV classification [8]. A multi-national study of 220 patients observed that the prevalence of depression remained as high as 33% for up to 5 years post stroke [9]. In addition, a review assessing post-stroke mortality reported increased odds of mortality for a period of 2 to 5 years among patients with depressive symptoms based on findings from 13 studies including 59,598 patients with stroke [10]. Previous research has suggested that the prevalence of depression and anxiety among carers is comparable to the observed prevalence levels among stroke survivors, and directly related to the severity of stroke [11–13]. In addition, there is also evidence that stroke survivors and their carers mutually influenced each other’s emotional state [14, 15].

Researchers have tested the benefits of using psychological interventions in the prevention as well as treatment of depressive symptoms for stroke survivors. Two Cochrane reviews assessed the benefits of psychological interventions for prevention [16] and for treatment of depressive symptoms [17] among stroke survivors. The first review concluded that there was a small but significant effect in preventing depressive symptoms in stroke patients (odds ratio 0.64; 95% confidence intervals 0.42 to 0.98) [16]; however, the second found no benefit in improvement of depressive symptoms, on the basis of three trials and 445 stroke patients [17]. Neither of these reviews identified any evidence of benefits of psychological interventions in stroke recovery nor suggested that further research is needed in this area [16, 17].

Mindfulness-Based Stress Reduction (MBSR) was developed by Jon Kabat-Zinn in North America in the 1980s. MBSR is a complex intervention and characteristically teaches a combination of mediation practices such as breath awareness, body awareness and awareness of movement through Hatha Yoga postures, alongside psychoeducational material on stress reduction (Kabat-Zinn 1990). Several systematic reviews and meta-analyses support the use of mindfulness-based interventions (MBIs) (largely derived from MBSR) in helping people with long-term conditions (LTCs) to cope better with improvements in symptoms of anxiety and depression [18–22]. A recent systematic review of the benefits of MBIs among patients with stroke, including four studies and 160 participants in total, concluded that a range of benefits may be derived from MBIs in this population, but cautioned that included studies had various limitations, such as small sample size, poor methodological quality and short follow-up duration [23]. Hence, large-scale, robust randomised controlled trials (RCTs) are needed before recommending integration of MBIs into routine clinical practice [23]. When testing complex interventions such as MBSR in novel populations and/or settings, participants’ experience on the acceptability and accessibility of the intervention can be seen as a necessary precursor for intervention optimisation before proceeding to large-scale trials [24].

### Aims and objectives

The aim of this study was to determine the feasibility of delivering MBSR to stroke survivors and their caregivers in the community. In view of the interdependence of depression among stroke survivors and caregivers, caregivers were also included in the development work for this intervention. The other objective was to assess acceptability of the standard MBSR practices among participants.

## Methods

### Study design

**Fig. 1:**
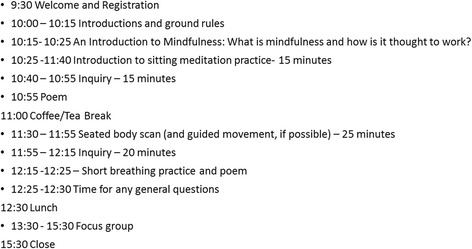
Study design schedule

The University of Glasgow Medical Veterinary and Life Sciences College Ethics Committee granted ethical approval for conducting this study (reference number 200150071). This was an observational feasibility study of delivering MBSR taster sessions to stroke survivors and caregivers in an urban community in Scotland (UK). Study participants attended one-off MBSR taster sessions in a group at a venue with facilities for disabled access. MBSR is based on meditation techniques and has three core practices—mindfulness of breathing (sitting meditation practice), mindfulness of the body (body scan) and mindful movement. Because many of those attending had mobility impairments, mindful movement was omitted from the taster session. Following each meditation, there is typically an ‘inquiry’ led by the facilitators as to how the participants experienced the session. Two experienced mindfulness practitioners (a medical doctor and a psychologist) delivered a 2-h MBSR taster session. The taster session was followed by focus group sessions conducted by the study team (please see Fig. 1).

### Recruitment

Participants were recruited from the community using newspaper adverts, social media postings and contacting patient-support groups run by local stroke-related charities over a period of 4 to 6 weeks, using convenience-sampling methods. The study inclusion criteria were having suffered from stroke at any time or being a carer for a stroke survivor, agreement to take part in the study and willingness to attend the study venue. Participants who had experienced a transient ischaemic attack (TIA) (without experiencing stroke) were excluded. Participants interested in taking part in the study were advised to contact the study team. Following this, a study information leaflet was posted to those expressing an interest, along with a consent form. Participants were contacted after 1 week via telephone to answer queries, if any. Participants wanting to go ahead were advised to sign the consent form and return them in the post. Participants recruited to the study were advised to attend the study on one of the two study dates. Caregivers or partners of all recruited participants were also offered study participation, wherever applicable. Please see Additional file 1 for recruitment protocol, participant information sheet and consent form used for recruitment.

### Data collection and analysis

On the day they attended to take part in the study, participants completed a questionnaire that included information on demographics, their diagnosis of stroke and other health problems. The questionnaire also included three self-rated symptom scores: Geriatric Depression Scale (GDS-15) [25], Zung’s self-rating scale for anxiety [26] and the Perceived Stress Scale (PSS) [27]. The participant questionnaire is described in detail in Additional file 2. We used a cut-off score of ≥ 6 for GDS-15 [25] and a score of ≥ 45 for Zung’s self-rating scale for anxiety, to define caseness [26]. These rating scales have been previously validated for use among stroke survivors [28–30].

**Fig. 2:**
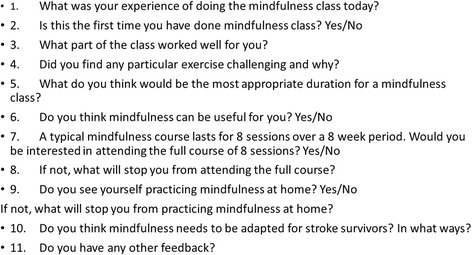
Focus group interview guide and questionnaire

Participants were given a feedback questionnaire after the taster sessions along with the interview guide prior to the focus groups. Three different members of the research team moderated two separate focus groups on each of the two study days (four focus groups in total). The focus groups were audio recorded and transcribed verbatim. Please see Fig. 2 for the interview guide and focus group questionnaire.

The data from the transcripts were analysed using an inductive approach guided by the framework analysis method [31]. Two members (BDJ and RS) of the research team independently read all the transcripts to immerse themselves in the data and develop a coding framework. A final coding framework was agreed with consensus among the research team. At least two researchers independently coded each transcript using the coding framework (BDJ coded all the transcripts, and another member from the research team independently coded each transcript). Differences in the coding between the researchers were resolved by consensus after discussion.

## Results

### Participant recruitment and characteristics

In response to the recruitment drive outlined above, 43 participants contacted the research team expressing interest in taking part. Five participants were excluded from the study due to either being ineligible (not stroke survivors or their caregivers) or due to their inability to attend the study centre due to the distance involved. Informed written consent was obtained from 38 participants before the study commenced. Ten participants withdrew their consent, as neither of the study dates offered were suitable. A total of 28 (21 stroke survivors and seven partners/caregivers) took part in the two study days; 16 on day 1 (11 stroke survivors and five carers) and 12 (10 stroke survivors and two carers) on day 2. All 28 participants took part in a focus group; two focus groups were held on each of the 2 days, which included eight, eight, six and six participants, respectively.

The study sample included 12 males (10 stroke survivors; two carers) and 16 females (11 stroke survivors; five carers). Half of the participants were over 60 years of age (*n* = 14). Out of the 21 participants with stroke, the majority (*n* = 15; 71.4%) regarded themselves as being disabled due to their stroke. Six participants (all stroke survivors) (21.4%) reported using a stick as a walking aid, while one participant (3.6%) was a wheelchair user. No participants reported communication difficulties. Six participants (five stroke survivors; one carer) (21.4%) reported having previous experience of mindfulness prior to study participation. The overall data completion rate was good (27/28; 97.4%), with only one participant not completing one of the three self-rating scales. Table 1 describes demographic- and health-related information for all participants.

**Table 1:** Study participant characteristics

Characteristics	*N* = 28 (21 stroke survivors; 7 carers/partners)	
	Stoke survivors	Carers
Female	12/21 (57.1%)	4/7 (57.1%)
Age in years	Median = 57 (IQR = 50 to 63); Mean = 56.3 (SD = 10.9)	Median = 64 (IQR = 64 to 67.5); Mean = 62.7 (SD = 12.6)
Current smoker—yes	2/21 (9.5%)	0/7
Currently employed—yes	4/21 (19%)	0/7
Years since stroke^a^	Median = 5 years (IQR = 2 to 7 years)	Not applicable
Number of self-reported health conditions (other than stroke)	Median = 2 (IQR = 2 to 4) Mean = 2.7 (SD = 1.6)	Median = 1 (IQR = 1 to 2); Mean = 1.6 (SD = 0.8)
Geriatric Depression Scale (GDS-15)	Median = 7 (IQR = 6 to 7); Mean = 6.6 (SD = 2)	Median = 5 (IQR = 5 to 6); Mean = 5.6 (SD = 1.4)
GDS-15 ≥ 6	17/21 (80.1%)	2/7 (28.6%)
Zung’s Self-Rating Anxiety Scale	Median = 38 (IQR = 28.7 to 45.2); Mean = 37.9 (SD = 9.8); missing values = 1	Median = 26 (IQR = 25 to 35); Mean = 32 (SD = 10.34)
Zung’s Self-Rating Anxiety Scale ≥ 45	6/21 (28.6%); missing values = 1	1/7 (14.3%)
Perceived Stress Scale	Median = 21(IQR = 18 to 25); Mean = 21 (SD = 5.1)	Median = 23 (IQR = 20.5 to 25); Mean = 23.4 (SD = 4.6)
Reported taking antidepressants	5/21 (23.8%)	0/7

*IQR* interquartile range, *SD* standard deviation

^a^Years since stroke reported for stroke survivors only (*n* = 21)

More than two thirds of the participants (67.8%) reported having depressive symptoms which met the threshold criteria for major depressive disorder (MDD), based on GDS-15 ≥ 6. A quarter of the study participants reported having significant anxiety symptoms, based on Zung’s rating scale ≥ 45. The mean PSS score, which measures psychological stress, was 21.7 among the study participants. The summary measure for depressive and anxiety symptoms was higher among stroke survivors, while perceived stress was reported to be higher among caregivers.

### Feedback questionnaire

The majority of the participants thought that mindfulness could be useful for them, while one participant thought it will not be useful and two participants were ‘not sure’. While three quarters of the study participants showed willingness to attend a full mindfulness course of eight sessions, six participants were not interested and one participant was ‘not sure’. Finally, apart from two participants (one each responded ‘no’ and ‘not sure’), all other participants thought that they could practice mindfulness at home (see Table 2).

**Table 2:** Feedback from participants

Feedback questions	Participants’ response—*N* = 28 (21 stroke survivors; 7 carers/partners)	
	Stroke survivors	Carers
Do you think mindfulness can be useful for you?	Yes 19/21 (90.5%)	6/7 (85.7%)
Would you be interested in attending the full mindfulness course of eight sessions?	Yes^a^ 16/21 (76.2%)	5/7 (71.4%)
Do you see yourself practicing mindfulness at home?	Yes 20/21 (95.2%)	6/7 (85.7%)

Focus group—themes identified: four main themes and nine sub-themes were identified from the data (Table 3)

^a^*N* = 4 participants responded with ‘yes’ but mentioned that they would only attend if the full course was made available locally

### Mindfulness experience

The majority of the participants reported an overall positive experience of the MBSR taster sessions. The participants frequently described the MBSR sessions as engaging and relaxing:

*Found it interesting. Very informative, and very relaxing (focus group 1, participant 2, stroke survivor)*

The participants noted experiencing different types of emotional responses after the MBSR taster sessions, ranging from hope to sadness:

*It’s very encouraging, you know…. And, actually, I’m filled with hope (focus group 2, participant 4, stroke survivor)*

*Well, sometimes, poetry can affect you in many ways, and, yes, it made me very sad, one of them, so… (focus group 3, participant 1, stroke survivor)*

Participants found it challenging to maintain focus and concentration during the MBSR exercises, but at the same time, a few participants recognised that it might help them to control their negative thoughts:

*I have nae (have no) focus, you’ve got to be very focused. It’s actually very hard to do, that’s the impression I get (focus group 1, participant 5, stroke survivor)*

**Table 3:** Emerging themes from focus group interviews

Main themes	Sub-themes
MBSR experience	Overall experience of MBSR
	Experience of different MBSR components
Organisation of MBSR sessions	Duration, frequency and timing (in relation to stroke) of MBSR sessions
	MBSR teacher experience and attributes, venue location and quality
Group and carer involvement	Advantages and disadvantages of group involvement
	Advantages and disadvantages of carer involvement
Future MBSR participation	Barriers and facilitators to future MBSR participation
	Suggestions for modifications to MBSR for stroke survivors
	Perceived purpose of mindfulness

*MBSR* Mindfulness-Based Stress Reduction

*Even things like the little button which I was focussing on something that, it was a good way of taking out everything which was not necessary. I thought that was good (focus group 4, participant 2, stroke survivor)*

Many participants reported a pleasant experience with the body scan component of the MBSR taster session:

*I particularly enjoyed the second one, ‘Body-scan’ (focus group 2, participant 1, stroke survivor)*

Some negative experiences related to the use of poetry and the use of bells to end the practices were reported by the participants:

*The one thing that I didn’t like, but this is part of mindfulness, I know, is the ringing of the bell to say the session’s at the end. I actually found that brought me out of the, sort of, relaxed state too fast. I’d have rather come out of it more slowly (focus group 2, participant 3, stroke survivor)*

*I found the poetry quite boring actually (focus group 3, participant 4, stroke survivor)*

### Organisation of MBSR sessions

A few participants felt that the duration of the MBSR taster session (2 h) was quite long and would have preferred a shorter duration:

*If we’re talking about 2 hours of mindfulness practice then yeah, it’s too much (focus group 2, participant 6, carer)*

Participants’ opinion on the timing of MBSR sessions in relation to a stroke event were varied, perhaps depending on level of impairment:

*If someone feels they’re physically able, then they might need it (MBSR) to catch the anxiety and the more psychological components of it (associated with stroke). For someone else who physically maybe needs rehab, physical rehab first, then maybe it’s further down… (focus group 3, participant 1, stroke survivor)*

Participants valued the attributes of the MBSR practitioners (a medical doctor and a psychologist):

*I think the way that it was run today, the feedback for me was, it was great, it was – from people that clearly have experience and know what they’re, they know what they’re talking about and, yeah, I found it very encouraging (focus group 2, participant 4, stroke survivor)*

### Group and carer involvement

Most participants suggested that group involvement could be a source of motivation and support:

*It is quite good to get other people’s (other stroke survivors) experiences (of living with stroke) and to know their opinions (focus group 4, participant 7, stroke survivor)*

However, some participants expressed concern that confidentiality was a key issue for participants to be able to express themselves:

*It’s what happens in a meeting stays in a meeting. It’s important that people understand that. And it’s (confidentiality is) implied and understood. And that (confidentiality) may help people to feel more open about that (expressing their feelings) (focus group 2, participant 2, stroke survivor)*

Participants welcomed the opportunity for carers/partners to take part along with stroke survivors:

*Some people wouldn’t be able to come if they didn’t have the carer, ‘cause some people need that carer with them pretty much the whole time (focus group 3, participant 3, stroke survivor)*

### Future mindfulness participation

Participants perceived the purpose of doing MBSR to facilitate relaxation and help with controlling emotions and viewed that as a facilitator for future participation in a full 8-week course:

*That means that’s giving me a wee bit o’ me-time (a small amount of my time), you know, just to get away from everybody and see the ones (thoughts) that are giving me a rough time, just block them oot (block them out) (focus group 1, participant 3, stroke survivor)*

Participants viewed the possibility of practicing mindfulness at any convenient place and time favourably

*Mindfulness is not golf. You don’t have to go and find a bag (golf bag) and, then, however many clubs to stick it out. And, then, find a membership (of a golf club) and then book a tee time it’s... you can do it anywhere. And, it’s (mindfulness) not, it’s not expensive. That’s another part of it (mindfulness), it’s accessible (focus group 2, participant 6, carer)*

Impairment from stroke, travel distance involved and uncertainty about doing the mindfulness techniques correctly were regarded as potential barriers to future MBSR participation:

*It’s (mindfulness is) going to be difficult for some people (stroke survivors), especially, you know, everyone’s got different impairments (due to stroke) and, but it (mindfulness) will have an effect (focus group 3, participant 2, stroke survivor)*

*But I still think at home you wouldn’t do it (mindfulness) as well (focus group 4, participant 4, carer)*

*Because if you look at it (mindfulness venue) on a map, and say, it’s there. But if you’ve got to go into Glasgow and come all the way out again, it’s not easy (travelling is not easy) if you’ve had a stroke (focus group 3, participant 5, stroke survivor)*

Finally, participants recommended the importance of gaining familiarity with the MBSR instructors and other participants (including the level and nature of their disability, if any) prior to starting the course. A pre-course orientation session was regarded as potentially beneficial:

*Just a one-to-one session just before the group starts where you just…, the person delivering (mindfulness to) the group is able to see what level of disability (due to stroke)? (focus group 3, participant 4, stroke survivor)*

*So, if you were holding a session for mindfulness for stroke survivors, it’s setting that scene and holding that space uniquely for people in that context. And, I think some of that is recognising that everybody is coming with different challenges with diff—either at a different place on their healing journey (after stroke) and there’s lots of different things (focus group 2, participant 3, stroke survivor)*

## Discussion

The aim of this study was to assess the feasibility of recruiting and delivering MBSR to stroke survivors and caregivers in the community. The study was able to recruit participants from the community using newspaper adverts, local patient support groups and social media resources. A total of 28 participants (21 stroke survivors and 7 partners/caregivers) took part in a one-off mindfulness taster session, followed by four separate focus groups. Prevalence of self-reported depression, anxiety and perceived stress was high among the participants. In addition, all stroke survivors who took part in the study regarded themselves as having some impairment due to stroke.

The majority of the study participants reported a positive experience with the MBSR taster sessions, showed willingness to take part in a full MBSR course and practice mindfulness at home. However, some participants suggested that local availability would be an important factor in their decision-making for attending a full MBSR course. Participants in this study found MBSR quite relaxing and viewed group involvement as a means of peer support and motivation. Many participants suggested that they would prefer a shorter MBSR session than the standard two and half hours. The potential of therapeutic gains (in controlling negative thoughts and emotions) from MBSR was viewed as a facilitator to taking part, while stroke-related disability and travel were viewed as barriers. An orientation session prior to the MBSR course was suggested as a means of developing familiarity and shared understanding between MBSR instructors and participants.

This is the first study to our knowledge that has collected qualitative data from stroke survivors and caregivers on any MBI. Three systematic reviews have explored the benefits of MBIs among stroke survivors [23, 32, 33] and have identified four original studies [34–37]. Three out of these four studies used MBSR, while one used mindfulness-based cognitive therapy (MBCT), a derivative of MBSR, tailored to treating people with recurrent depression [34–37]. These studies did not explore the views of participants on acceptability and accessibility of MBIs among stroke survivors [34–37]. The mean PSS score, which measures the mean age of participants in our study was approximately 56 years while the mean age of stroke survivors in the Western world has been reported as 75 years [38]; our sample may not be representative of average stroke survivors. Psychological stress was 21.7 among the study participants, which is higher compared to means PSS score reported from a general population sample [39]. Participants in this study found MBSR quite relaxing and they viewed group involvement as a means of peer support and motivation. Previous qualitative studies on MBSR for coronary heart disease patients, diabetes patients and breast cancer survivors have reported similar findings [40, 41]. An orientation session and a shorter duration were recommended as possible MBSR modifications in this study. MBI modifications have been successfully implemented by other studies in a healthcare setting for other health conditions such as multiple sclerosis and drug addiction [42, 43].

The key strengths of this study are collection of good qualitative data on user experience for MBI among stroke survivors and their carers and good data completion rate. This study has some limitations. Participants in this study were self-selected and they initiated contact with the research team after reading the study advert. We did not assess cognitive capacity while consenting participants for the study. The study was offered only in one geographical location, and we did not have sufficient resources to provide funds for transport. This may have introduced a selection bias. A one-off MBSR taster session was provided for two groups, but participants were not followed up after these sessions. Therefore, it is not possible to comment on acceptability of a full 8-week MBSR course here or their willingness to take part in a randomised controlled trial involving MBSR. In addition, it was not possible to explore the acceptability or accessibility of standard MBSR home practices among participants. Finally, the median duration since stroke episode for the study participants was 5 years while stroke survivors are likely to experience more severe depressive symptoms in the early period following their stroke [7, 8], which may act as a potential barrier in their participation in a group-based activity such as MBSR.

## Conclusion

It was feasible to recruit 21 stroke survivors and seven caregivers from the community (using newspaper adverts and stroke support groups) to take part in MBSR taster sessions. The prevalence of self-reported depressive symptoms, anxiety and perceived stress was high among participants. MBSR taster sessions were well received by most participants, with the majority expressing willingness to take part in a future full course. Peer support and caregiver involvement were viewed positively by most of the participants. Potential for therapeutic gains was the perceived facilitator for future MBSR participation, while stroke-related impairment and travel involved were perceived as potential barriers. Shorter duration of MBSR sessions and an initial orientation session prior to the course were suggested as MBSR modifications for stroke survivors. The findings from this study should be taken into consideration while developing any future MBI to cater for stroke survivors and/or their caregivers. The acceptability and benefits of making these minor modifications need to be tested in a feasibility study offering a full 8-week MBSR course for stroke survivors and/or their caregivers. A feasibility study exploring the acceptability of a full 8-week MBSR course for stroke survivors and/or caregivers is needed.

## Additional files

Additional file 1: Mindfulness in Stroke – Participant Recruitment Protocol. (PDF 649 kb)

Additional file 2: Mindfulness in Stroke Study. (PDF 313 kb)

## Abbreviations

GDS-15: Geriatric Depression Scale
IQR: Interquartile range
MBSR: Mindfulness-Based Stress Reduction
PSS: Perceived Stress Scale
SD: Standard deviation
