# Supplementary material for: Acceptability of mindfulness from the perspective of stroke survivors and caregivers: a qualitative study
Source: Pilot Feasibility Stud. 2018 Feb 26;4:57. doi: 10.1186/s40814-018-0244-1 (PMC5827989; doi:10.1186/s40814-018-0244-1)
Supplement: Supplementary file 1 — Mindfulness in Stroke – Participant Recruitment Protocol. (PDF 649 kb) [file 40814_2018_244_MOESM1_ESM.pdf]

Mindfulness in Stroke – Participant Recruitment Protocol

Two different dates

Step1: Interested participant contact BJ via phone/e-mail

Step 2: BJ to check eligibility on phone:

1. Exclude TIA- Yes
2. Ability to attend ALLIANCE venue- Yes

Name:

Address:

Patient/Carer:

Contact Number:

E-mail:

When was the stroke?

Any disability of note?

How did you find out about the study?

Step 3: Send Participant Information Leaflet (PIL) and consent form in post with a stamped return envelope.

Consent form and PIL posted?

Step 4: Participant who sign and return consent form to be added to the project participant list.

Consent form received?

University of Glasgow | College of Medical, Veterinary & Life Sciences

PARTICIPANT INFORMATION SHEET

1. **Study title**

Exploring the role of mindfulness based stress reduction in stroke survivors in community- A development study

2. **Invitation paragraph**

You are being invited to take part in a research study exploring the use of Mindfulness-based stress reduction (MBSR) meditation techniques for people who have survived a stroke. Before you decide it is important for you to understand why the research is being done and what it will involve. Please take time to read the following information carefully and discuss it with others if you wish. Ask us if there is anything that is not clear or if you would like more information. Please take time to decide whether or not you wish to take part.

3. **What is the purpose of the study?**

Mood disturbances are very common occurrence in Stroke survivors with majority of them experiencing low mood, anxiety and fatigue. Talking therapies have been found to have some benefit in treating these symptoms for stroke survivors. MBSR works in a different way from standard talking therapies and may be more or less lasting in its effects than talking therapies. The purpose of this study is to offer “taster sessions” for MBSR to stroke survivors and get feedback from participants on their experience, including possible suggestions to modify MBSR for stroke survivors.

4. **Do I have to take part?**

No, you do not have to take part in this study. It is up to you to decide whether or not to take part. If you do decide to take part, you will be given this information sheet to keep and be asked to sign a consent form. If you decide to take part, you are still free to withdraw at any time and without giving a reason. Participants may withdraw from participation at any stage without any reason and without any consequences.

5. **What will happen to me if I take part?**

If you agree to take part in this study, you will have the opportunity to take part in MBSR taster sessions and subsequent focus group interviews. You will be invited to attend the site

of MBSR in a community setting, somewhere in Glasgow **(location- ALLIANCE, Venlaw Building**
349 Bath Street, Glasgow, G2 4AA). You will be attending the session in a group of up to 20 participants. The participants will be expected to complete a brief questionnaire which will include their demographics and medical history details. It will take approximately 20-30 minutes for the participants to complete the questionnaire. You will take part in two MBSR taster sessions (each lasting for 45 mins-1 hour with a break between two sessions) which will last for 2.5 hours in total. The MBSR taster sessions will be delivered by fully qualified MBSR practitioners.

The MBSR taster sessions will be followed by a lunch break. After lunch, they will be invited to take part in a focus group interviews (along with other participants) which will be conducted by one of the members of project team. The focus group interviews will last approximately last for 1 hour. In total, participants will be expected to be present at the site between 09:30 am and 3:30 pm on one day. The participants will not be expected to take part in any other research related activities after that. We will aim to recruit one "expert participant" who would advise us on the findings of the study and any future developments.

6. **What do I have to do?**

You do not have to make any changes to your lifestyle or to your medications if you take part in the study. You will be advised to behave as you normally would on the day of the taster sessions.

7. **What are the possible disadvantages and risks of taking part?**

The only theoretical risk of taking part is that MBSR might make some people more aware of their feelings. When someone is anxious or experiencing low mood, this might make the feelings more intense. If this happens, you will be offered the chance to debrief with the course facilitator, who will be a very experienced MBSR practitioner.

8. **What are the possible benefits of taking part?**

The information that is collected during this study will give us a better understanding of stress management therapies for stroke survivors and will inform future development of MBSR therapies tailored for stroke survivors. Participants may also find that the MBSR helps with their own management of stress.

9. **Will my taking part in this study be kept confidential?**

All information collected about you during the course of the research will be kept strictly confidential. You will be identified by an ID number. Any information about you will have your name and address removed so that you cannot be recognised from it. You will be invited to take part in a focus group interview (along with other participants); it will be recorded, and will thereafter be transcribed by departmental administrative staff. All transcriptions will be rendered anonymous and every effort will be made to protect your anonymity.

Your anonymised information will be kept by the University of Glasgow for 10 years before being destroyed, in keeping with the University policies.

10. **What will happen to the results of the research study?**

All participants will be provided with a written lay summary of the findings from this study. You will not be identified in any report/publication. However, the results or the project report summary will be written up and will be publicly available at the time of completion (Oct 2016). The findings will also be written up in an academic journal article format, once all of the data has been analysed.

11. **Who is organising and funding the research?**

This research has been organised by researchers in the University of Glasgow, Institute of Health and Wellbeing, Department of General Practice, and has been funded by the R S Macdonald Charitable Trust.

12. **Who has reviewed the study?**

The project has been reviewed by the University of Glasgow, Medical Veterinary and Life Sciences College Ethics Committee.

13. **Contact for Further Information**

Dr Bhautesh Jani
Bhautesh.jani@glasgow.ac.uk
General Practice and Primary Care
University of Glasgow
1 Horselethill Road
Glasgow G12 9LX
01413308353

14. Thank you for reading this information sheet and expressing your interest in this study.

University of Glasgow | College of Medical, Veterinary & Life Sciences

Centre Number:
Project Number:
Subject Identification Number for this trial:

CONSENT FORM

Title of Project:

Exploring the role of mindfulness based stress reduction in stroke survivors in community- A development study

The study is funded by the R S MacDonald Charitable Trust; under the grant name "Seedcorn funding for Living Well with Multimorbidity".

Name of Researcher(s):

Dr Bhautesh Jani
Dr Maggie Lawrence
Dr Robert Simpson
Prof Stewart Mercer

Please initial box

I confirm that I have read and understand the information sheet dated ___________ (______) for the above study and have had the opportunity to ask questions. ☐

I understand that my participation is voluntary and that I am free to withdraw at any time, without giving any reason, without my legal rights being affected. ☐

I agree to take part in the above study. ☐

______________________	__________	______________________
Name of participant	Date	Signature
______________________	__________	______________________
Researcher	Date	Signature
