# Supplementary material for: Acceptability of mindfulness from the perspective of stroke survivors and caregivers: a qualitative study
Source: Pilot Feasibility Stud. 2018 Feb 26;4:57. doi: 10.1186/s40814-018-0244-1 (PMC5827989; doi:10.1186/s40814-018-0244-1)
Supplement: Supplementary file 2 — Mindfulness in Stroke Study. (PDF 313 kb) [file 40814_2018_244_MOESM2_ESM.pdf]

Mindfulness in Stroke Study

Please take your time to complete this questionnaire. Please contact one of the research team members if you need help with answering these questions. Thank you!

Section 1: Participant Information

Q1Could you please provide your full name, your full postal address, your contact phone number if possible?

Q2 how did you find out about this study? Eg. Metro Advert

Q3 Please state you age in years and your gender.

Q4 Please state your ethnicity

Q5 Do you currently smoke?

Yes or No

Q6 Are you currently in employment?

Yes or No

Q7 Can you please state your educational qualifications?

Section 2 Participant Health Status

This section has various questions on your health status. If you are with your partner who had stroke, go to Q4.

Q1 Please state the year in which you were diagnosed with Stroke (for e.g. 2016).

Q2 Have you had more than one episode of Stroke or TIA?

Yes/No

If yes, please go to Q3, if no, please go to Q4.

Q3 How many episodes of Stroke or TIA (often called as “mini stroke”) have you suffered from? Please state the year next to each episode (for e.g. 1st episode: Stroke-2011, 2nd episode: TIA-2015)

Q4 what medications you are currently taking on a regular basis?

Q5 Would you describe yourself as having a disability?

Yes or NO

If yes, please go to Q 6 and Q7. If not, please go to Q8.

Q6 Is your disability related to one or more of your stroke events?

Yes or No

Q7 How would you describe your disability?

Q8 **Has a doctor ever told you that you had any of the following?**

please √ the first box for every condition you have(including stroke and mini stroke), and rate in the second box each condition in relation to how much it <u>currently</u> limits your daily activities from on a scale from 1 (NOT AT ALL) to 5 (A LOT).

**High Blood Pressure**	☐☐	**Arthritis**	☐☐	**Asthma**	☐
**Stroke / mini-stroke**	☐☐	**Back Problems**	☐☐	**Chronic Bronchitis**	☐
**Diabetes**	☐☐	**Thyroid Problem**	☐☐	**Migraine**	☐
**Angina /Heart Attack**	☐☐	**Eczema / Psoriasis**	☐☐	**Cancer**	☐
**Heart Failure**	☐☐	**Liver Disease**	☐☐	**Irritable Bowel Syndrome**	☐
**Anxiety / Depression**	☐☐	**Kidney Disease**	☐☐		

Other (please state) ____________________

Section 3 Your Mood – based on GDS 15

This section has various questions to assess how your mood has been in recent weeks.

Part 1- Low mood symptoms

Choose the best answer for how you have felt over the past week:

1. Are you basically satisfied with your life? YES / NO
2. Have you dropped many of your activities and interests? YES / NO
3. Do you feel that your life is empty? YES / NO
4. Do you often get bored? YES / NO
5. Are you in good spirits most of the time? YES / NO
6. Are you afraid that something bad is going to happen to you? YES / NO
7. Do you feel happy most of the time? YES / NO
8. Do you often feel helpless? YES / NO
9. Do you prefer to stay at home, rather than going out and doing new things? YES / NO
10. Do you feel you have more problems with memory than most? YES / NO
11. Do you think it is wonderful to be alive now? YES / NO
12. Do you feel pretty worthless the way you are now? YES / NO
13. Do you feel full of energy? YES / NO
14. Do you feel that your situation is hopeless? YES / NO
15. Do you think that most people are better off than you are? YES / NO

Part 2 Anxiety symptoms (based on Zung's Self Rating Scale)

For each item below, please place a check mark (√) in the column which best describes how often you felt or behaved this way during the past several day.

Place check mark (√) in correct column

	A little bit of time	Some of the time	Good part of time	Most of the time
I feel more nervous and anxious than usual				
I feel afraid for no reason at all.				
I get upset easily or feel panicky.				
I feel like I'm falling apart and going to pieces.				
I feel that everything is all right and nothing bad will happen.				
My arms and legs shake and tremble.				
I am bothered by headaches neck and back pain.				
I feel weak and get tired easily.				
I feel calm and can sit still easily.				
I can feel my heart beating fast.				
I am bothered by dizzy spells.				
I have fainting spells or feel like it.				
I can breathe in and out easily.				
I get feelings of numbness and tingling in my fingers & toes.				
I am bothered by stomach aches or indigestion.				
I have to empty my bladder often.				
My hands are usually dry and warm.				
My face gets hot and blushes.				
I fall asleep easily and get a good night's rest.				
I have nightmares.				

Part 3

Perceived Stress Scale

The questions in this scale ask you about your feelings and thoughts during the last month. In each case, you will be asked to indicate by circling how often you felt or thought a certain way

0 = Never, 1 = Almost Never, 2 = Sometimes, 3 = Fairly Often, 4 = Very Often.

In the last month, how often have you been upset because of something that happened unexpectedly?................................ 0 1 2 3 4.

In the last month, how often have you felt that you were unable to control the important things in your life? .... 0 1 2 3 4.

In the last month, how often have you felt nervous and "stressed"? .... 0 1 2 3 4.

In the last month, how often have you felt confident about your ability to handle your personal problems? .... 0 1 2 3 4.

In the last month, how often have you felt that things were going your way?... 0 1 2 3 4.

In the last month, how often have you found that you could not cope with all the things that you had to do? .... 0 1 2 3 4.

In the last month, how often have you been able to control irritations in your life?... 0 1 2 3 4.

In the last month, how often have you felt that you were on top of things?.. 0 1 2 3 4.

In the last month, how often have you been angered because of things that were outside of your control?.................................. 0 1 2 3 4.

In the last month, how often have you felt difficulties were piling up so high that you could not overcome them? .... 0 1 2 3 4.
